# Can epilepsy be predicted after the first febrile seizure? Insights from machine learning of postictal EEG

**DOI:** 10.1002/epd2.70250

**Published:** 2026-04-29

**Authors:** Boran Şekeroğlu, Hüseyin Öztoprak, Özlem Yayıcı Köken, Hatice Demir, Mehpare Sarı Yanartaş, Deniz Yılmaz, Burçin Şanlıdağ, Ayşegül Neşe Çıtak Kurt

**Affiliations:** ^1^ Department of Computer Engineering Faculty of AI and Informatics, Near East University Nicosia Türkiye; ^2^ Signal and Communication Technologies Lab, Department of Electrical and Electronics Engineering Cyprus International University Nicosia Cyprus; ^3^ Division of Pediatric Neurology, Department of Pediatrics, Faculty of Medicine Akdeniz University Antalya Türkiye; ^4^ Department of Pediatrics Yenimahalle State Hospital Ankara Türkiye; ^5^ Division of Pediatric Neurology, Department of Pediatrics Ankara Bilkent City Hospital Ankara Türkiye; ^6^ Division of Pediatric Neurology, Department of Pediatrics, Faculty of Medicine Near East University Nicosia Cyprus; ^7^ Brain and Conscious States Research Center Nicosia Cyprus; ^8^ Division of Pediatric Neurology, Department of Pediatrics, Faculty of Medicine Yıldırım Beyazıt University Ankara Türkiye; ^9^ Present address: Department of Pediatrics İstanbul Maltepe Government Hospital İstanbul Türkiye; ^10^ Present address: İstanbul Göztepe Prof Dr Süleyman Yalçın City Hospital İstanbul Türkiye

**Keywords:** child, electroencephalography, epilepsy, febrile seizures, machine learning, prognosis

## Abstract

**Objective:**

Febrile seizures (FS) are the most common seizures in childhood, yet identifying children at risk of developing epilepsy after the first FS remains challenging. We aimed to evaluate the prognostic potential of machine learning (ML) algorithms applied to post‐febrile seizure electroencephalography (EEG) recordings.

**Methods:**

We retrospectively reviewed 104 children (69 boys; mean age at first febrile seizure: 39.4 ± 18.2 months) who presented with their first febrile seizure between January 2018 and December 2021. Clinical data and EEG recordings obtained during N2 sleep were collected. EEG analysis was performed using separate preprocessing pipelines. For conventional EEG analysis, recordings were band‐pass filtered between 1 and 40 Hz, and artifact‐free segments were analyzed using Python‐based pipelines (YASA, MNE) to extract 34 time‐domain. The 34 extracted electrophysiological features were calculated across different bipolar EEG channels and evaluated together with aggregated inter‐channel measures, resulting in a total of 93 input attributes used for ML model development. High‐frequency oscillations (HFOs) were analyzed using a distinct pipeline applied to wideband EEG data before low‐pass filtering. Six machine learning algorithms—J48 Consolidated Decision Tree, Random Forest, Gradient Boosting, Extreme Gradient Boosting, k‐nearest neighbor, and Support Vector Machine—were trained using 10 × 7 repeated cross‐validation. Model performance was evaluated using sensitivity, specificity, accuracy, area under the receiver operating characteristic curve (ROC AUC), and F1‐score.

**Results:**

Over a median follow‐up of 4.2 months, 13 patients (12.5%) developed epilepsy, and all diagnoses were made within 9 months. XGBoost achieved the highest accuracy (0.89) and specificity (0.95) but had low sensitivity (0.46). J48 achieved the highest sensitivity (0.87) and ROC AUC (0.79), with a specificity of 0.71. Incorporating clinical features, including recurrent seizures, increased sensitivity to 0.95. The most relevant predictors were patient history, frequency band power, particularly increased power in lower frequency bands, and high‐frequency oscillations counts.

**Conclusion:**

ML‐based analysis of initial EEG after a first febrile seizure may assist in early epilepsy risk stratification. J48 provided superior sensitivity, and combining electroencephalography‐derived biomarkers with clinical data further enhanced predictive performance. Prospective, multicenter studies are warranted to confirm these findings.

**Key points**

- Machine learning applied to postictal electroencephalography (EEG) may support early epilepsy risk stratification after a first febrile seizure.
- N2 sleep EEG provides stable conditions for extracting clinically relevant electrophysiological features.
- High‐frequency oscillations and patient history emerged as key predictors of future epilepsy risk.
- Models with higher sensitivity may be preferable for early risk stratification in imbalanced clinical datasets.
- External validation is required before machine learning models can be translated into routine clinical practice.

## 1 INTRODUCTION

Febrile seizures (FS) are the most common type of seizure in children without chronic neurological disorders.^1^ They typically occur between 6 months and 5 years of age, in the absence of central nervous system infection, metabolic disturbance, or a history of epilepsy.^2^ The incidence of FS ranges from 2 to 5% in most populations^3^, ^4^ with higher rates of 8–10% reported in Asian populations.^5^, ^6^

The pathophysiology of FS is multifactorial, involving both genetic and environmental factors.^7^, ^8^, ^9^ Elevated levels of inflammatory mediators, such as cytokines, have been associated with an increased risk of FS.^7^ These inflammatory responses may be influenced by genetic predisposition—particularly variants affecting sodium channels^1^, ^7^, ^8^ as well as specific infectious agents. Common viral triggers include influenza, adenovirus, parainfluenza virus, and human herpesvirus‐6.^10^, ^11^, ^12^

FS are classified as simple, complex, or febrile status epilepticus based on seizure duration, presence of focal features, recurrence within 24 h, and pre‐existing neurological abnormalities.^13^, ^14^, ^15^ Approximately 70% of cases are simple FS, 25% are complex FS, and 5% are febrile status epilepticus (FSE).^13^, ^16^, ^17^ This classification has prognostic implications: the lifetime risk of epilepsy is about 0.5% in the general pediatric population, 1–2% after a simple FS, and 6–8% after a complex FS.^18^, ^19^ Importantly, the lifetime risk of developing epilepsy after FSE is substantially higher, reaching approximately 10–20%, as reported in long‐term population‐based studies.^19^, ^20^ The predictive value of electroencephalography (EEG) after FS remains controversial.^20^ Some studies have reported that focal paroxysmal epileptiform discharges following a complex FS predict subsequent epilepsy^21^, ^22^, ^23^, ^24^ with 25–30% of such children later diagnosed with epilepsy.^25^, ^26^, ^27^ However, other studies have found no significant prognostic correlation. For example, Choudhari et al. reported an association between midline vertex discharges and complex FS, but no link with epilepsy risk.^21^

Despite established classification criteria,^7^ accurately predicting which children with FS will later develop epilepsy remains challenging. The present study aims to apply machine learning (ML) techniques to EEG data obtained after the first FS, to identify electrophysiological markers that could improve the prediction of future epilepsy. By integrating advanced computational methods into clinical assessment, we aim to enhance diagnostic precision and support better long‐term management of children experiencing their first FS.

## 2 MATERIALS AND METHODS

This study was conducted in accordance with the principles of the Declaration of Helsinki and approved by the Ankara City Hospital No. 1 Clinical Research Ethics Committee in 2021 (Approval Number: E2‐21‐96).

### 2.1 Study design and participants

This retrospective observational study included pediatric patients who presented with their first FS to the Pediatric Neurology Department between January 2018 and December 2021. Inclusion criteria were:

1. Age between 6 months and 5 years at seizure onset.
2. Diagnosis of FS according to International League Against Epilepsy (ILAE) criteria.
3. Availability of post‐FS EEG recording.

Exclusion criteria were:

1. Pre‐existing epilepsy.
2. Evidence of central nervous system infection or structural abnormality.
3. Known metabolic or genetic disorders.

Structural brain abnormalities were assessed using brain magnetic resonance imaging (MRI), which was performed in patients presenting with atypical clinical features, complex febrile seizures, or abnormal neurological examination findings. In patients with simple febrile convulsions and normal neurological examination, no MRI had been performed as it is not indicated. Central nervous system infection was excluded based on clinical evaluation, laboratory investigations, and cerebrospinal fluid analysis when clinically indicated. Metabolic and genetic disorders were evaluated based on detailed history and systemic‐ neurological examination. Children were tested for metabolic and genetic disorders selectively in the presence of clinical red flags, including developmental delay, dysmorphic features, abnormal neurological examination, or atypical seizure characteristics. Patients in whom an underlying metabolic or genetic etiology was identified were excluded from the study.

### 2.2 Data collection

Clinical and demographic data were extracted from patient medical records and the hospital electronic health system. Variables included sex, age at first seizure, family history of FS or epilepsy, seizure type (simple/complex), number of FS episodes, recurrence within 24 h, and long‐term outcomes (diagnosis of epilepsy, initiation of antiseizure drugs).

### 2.3 Dataset

A total of 104 patients met the inclusion criteria, including 69 boys (66.3%) and 35 girls (33.7%). A family history of FS was present in 43.5% of the cohort, and 12.1% reported a family history of epilepsy. The mean age at first FS was 39.4 months, with an average of 1.6 seizures per patient. Recurrent seizures occurred in 26.6% during follow‐up as shown in Table 1. Epilepsy was diagnosed in 12.5% of patients, and 9.7% required antiseizure medication. A subgroup of 12.9% had ≥2 years of follow‐up.

**TABLE 1:** Key demographic and clinical characteristics of the study population.

Variable	Value
Total number of patients (*n*)	104
Female patients, *n* (%)	35 (33.7%)
Family history of febrile seizures (%)	43.5%
Family history of epilepsy (%)	12.1%
Mean age at first seizure (months)	39.4
Mean total number of seizures	1.6
Recurrent seizures during follow‐up (%)	26.6%
Diagnosed with epilepsy during follow‐up (%)	12.5%
Initiated on antiepileptic treatment (%)	9.7%
Follow‐up ≥2 years (%)	12.9%

### 2.4 EEG preprocessing and feature extraction

EEGs were recorded after the first febrile seizure, typically within the early follow‐up period within 1–3 weeks. EEG data were acquired during N2 sleep. Before statistical analysis and ML classification, EEG preprocessing and feature extraction were performed using two methodologically distinct pipelines, depending on the type of electrophysiological features analyzed.

For conventional EEG analysis, including background rhythm characterization and frequency band–power features, recordings were band‐pass filtered between 1 and 40 Hz to remove slow drifts and high‐frequency noise. Line noise was attenuated using notch filtering and no resampling was applied. From these filtered signals, standard time‐domain and frequency‐domain features were extracted, including absolute and relative power across canonical frequency bands (delta, theta, alpha, sigma, beta, and gamma), as well as sleep spindle–related measures.

For high‐frequency oscillations (HFOs) analysis, a dedicated processing pipeline was applied to wideband EEG data (80–250 Hz) before conventional low‐pass filtering to preserve high‐frequency activity and prevent filtering‐induced artifacts. Raw EEG recordings were first visually inspected, and artifact‐free segments were selected. Common artifacts—including cardiac, ocular, movement‐related, and powerline interference—were identified and excluded using a combination of visual inspection and automated detection methods; independent component analysis was not applied. No low‐pass filtering at 40 Hz was applied before HFOs extraction, thereby avoiding methodological incompatibility and minimizing the risk of spurious high‐frequency activity arising from filtered sharp transients.

EEG signals were referenced using a standard clinical referencing scheme (linked mastoids) and segmented into artifact‐free N2 sleep epochs before feature extraction. EEG preprocessing and feature extraction were conducted using Python‐based analysis pipelines, primarily utilizing the YASA and MNE libraries.

For each patient, a total of 34 individual electrophysiological features were derived, encompassing frequency band–power measures, sleep spindle characteristics, and detailed HFOs metrics, including counts, durations, overlapping and non‐overlapping events, density, amplitude, and average frequency. Features were extracted from specific bipolar montage channels (Fz–Cz and Cz–Pz) as well as from aggregated multi‐channel data where appropriate. In total, these features yielded 93 attributes used as input variables for ML model training.

A detailed overview of all demographic and EEG‐derived features included in the analysis is provided in Table 2.

**TABLE 2:** Demographic and extracted time‐domain features.

Extracted and demographic features	
Gender	Delta^a^
Family (FK)	Theta^a^
Family (epilepsy)	Alpha^a^
FK Type	Sigma^a^
Fokal/Jeneralize	Beta^a^
Total # of seizures	Gamma^a^
Duration of first seizure	Total Abs. Power^a^
Total # of HFOs	Total Duration of HFOs^b^
Total # of sleep spindles	Average Duration of HFOs^b^
Number of overlapping HFO	Total Duration of Sleep Spindles
Overlapping HFO%	Average Duration of Sleep Spindles
Total duration of overlapping HFO	N2 Stage Duration
Average duration of overlapping HFO	Density
Number of non‐overlapping HFO	Average Amplitude
Non‐overlapping HFO%	Absolute Power
Total duration of non‐overlapping HFO	Relative Power
Average duration of non‐overlapping HFO	Average Frequency

^a^ Extracted for Fz‐Cz and Cz‐Pz montage channels separately.

^b^ Extracted for each montage channel separately (×18).

### 2.5 Machine learning models

Six ML algorithms were evaluated: four tree‐based models—J48 Consolidated Decision Tree (J48), Random Forest (RF), Gradient Boosting (GB), and Extreme Gradient Boosting (XGBoost)—and two non‐tree‐based models—k‐nearest neighbor (kNN) and Support Vector Machine (SVM). Tree‐based models were chosen for their interpretability and ability to capture complex nonlinear relationships, whereas kNN and SVM were included for comparative performance despite their lower interpretability.

- **J48 Minority Reweighted:** A variant of the C4.5 decision tree algorithm that selects attributes based on information gain derived from entropy measures.^28^ The minority reweighted implementation addresses class imbalance by virtually increasing minority class representation during training. Tree pruning was applied to remove non‐informative branches and reduce overfitting.
- **Random Forest (RF):** An ensemble of decision trees generated from bootstrap samples, with random feature selection at each split; predictions are aggregated via bagging to improve generalization.^29^
- **Gradient Boosting (GB):** A sequential ensemble method in which each new decision tree corrects the residual errors of the previous model using gradient‐based optimization.^30^
- **Extreme Gradient Boosting (XGBoost):** An optimized gradient boosting framework incorporating L1/L2 regularization and additional enhancements for robustness and speed.^31^
- **k‐Nearest Neighbor (kNN):** A distance‐based algorithm that classifies samples according to the majority label among their *k* closest neighbors.^32^
- **Support Vector Machine (SVM):** A margin‐based classifier that separates classes via an optimal hyperplane; kernels enable nonlinear decision boundaries.^33^

### 2.6 Experiments and evaluation metrics

#### 2.6.1 Experiments

All models were trained on demographic and EEG‐derived features using 10 × 7 repeated cross‐validation, ensuring that each observation served as both training and testing data across folds. Hyperparameters were optimized using grid search for each algorithm (Table 3).

**TABLE 3:** Optimal hyperparameters for each ML algorithm after grid search.

Algorithm	Optimal parameters
SVM	RBF kernel, *C* = 1, *γ* = 0.01
RF	n_estimators = 75
GB	Learning rate *η* = 0.07, n_estimators = 100
XGBoost	Learning rate *η* = 0.01, max_depth = 9
kNN	*k* = 3, Euclidean distance
J48 Minority Reweighted	Minority oversampling, pruning enabled

#### 2.6.2 Evaluation metrics

Model performance was evaluated using sensitivity, specificity, accuracy, area under the receiver operating characteristic curve (ROC AUC), and macroaveraged F1 score. These metrics were selected to account for class imbalance and to provide a balanced assessment of model performance.

## 3 RESULTS

Among 104 children with FS, 13 (12.5%) developed epilepsy during long‐term follow‐up, while the remaining subjects served as controls. Among the 13 children who developed epilepsy during follow‐up, the mean time interval between the first febrile seizure and the diagnosis of epilepsy was 4.2 months, ranging from 1 week to 9 months. Baseline demographic characteristics, including age at first febrile seizure and sex, did not significantly differ between children who developed epilepsy and those who did not. In contrast, seizure‐related clinical features, particularly recurrent febrile seizures, were more frequently observed in children who later progressed to epilepsy.

### 3.1 Model performance

Overall, Support Vector Machine (SVM), Extreme Gradient Boosting (XGBoost), and Gradient Boosting (GB) showed high specificity but low sensitivity, reflecting difficulty in correctly identifying positive cases in the imbalanced dataset. XGBoost achieved the highest accuracy (0.89) and specificity (0.95) but had limited sensitivity (0.46). Corresponding F1 and ROC AUC scores were 0.67 and 0.70, respectively. SVM and GB followed with accuracy scores of 0.87 and 0.83, and specificity of 0.92 and 0.89, but their sensitivity remained low (0.46 and 0.38).

Random Forest (RF) and k‐nearest neighbor (kNN) produced more balanced sensitivity values (both 0.61). RF achieved a specificity of 0.82 and outperformed kNN in F1 (0.67 vs. 0.52) and ROC AUC (0.71 vs. 0.62).

The J48 Minority Reweighted decision tree demonstrated the highest sensitivity (0.87), with moderate specificity (0.71) and stable performance across all metrics. It yielded the highest ROC AUC (0.79) and robust F1 score (0.61), outperforming other models in identifying positive cases. All performance metrics are presented in Table 4.

**TABLE 4:** Summary of all performance metrics.

MODEL	Sensitivity	Specificity	Accuracy	F1‐score	ROC AUC
SVM	0.46	0.92	0.87	0.63	0.68
RF	0.61	0.82	0.87	0.67	0.71
XGBoost	0.46	**0.95**	**0.89**	0.67	0.70
kNN	0.61	0.64	0.63	0.52	0.62
GradBoost	0.38	0.89	0.83	0.59	0.62
J48 Minority Reweighted	**0.87**	0.71	0.73	0.61	**0.79**

*Note*: Higher values are highlighted in bold to improve clarity.

### 3.2 Feature importance

Feature importance analysis revealed that clinical history variables and specific EEG‐derived features contributed most strongly to epilepsy risk classification. Among clinical parameters, recurrent febrile seizures emerged as the most influential predictor, consistently increasing the probability of epilepsy across models.

With respect to EEG‐derived features, increased power in lower frequency bands, particularly delta and theta activity, as well as higher counts and longer durations of HFOs, were among the strongest contributors to model predictions. These features were positively associated with epilepsy classification, indicating that higher values increased the likelihood of being classified into the epilepsy group.

In contrast, relatively preserved background activity and lower HFOs burden were more frequently associated with the non‐epilepsy group. The directionality of feature influence was most clearly interpretable in tree‐based models, particularly the J48 decision tree, allowing direct assessment of how individual clinical and electrophysiological features contributed to classification outcomes.

## 4 DISCUSSION

FS remain the leading cause of seizures in the pediatric population, yet predicting the risk of subsequent epilepsy after the first episode remains challenging due to the heterogeneity of clinical and electrophysiological profiles.^34^ In this retrospective study of 104 children with FS, we aimed to predict epilepsy development using six different ML algorithms—J48 Consolidated Decision Tree, Random Forest, Gradient Boosting, Extreme Gradient Boosting, k‐Nearest Neighbor, and Support Vector Machine—trained on 34 time‐domain EEG features extracted from N2 sleep stage recordings obtained after the initial seizure. The N2 sleep stage was specifically selected as it provides relatively stable electrophysiological conditions and has been shown to facilitate the detection of epileptiform discharges and oscillatory biomarkers, including HFOs. Among 104 children with FS, 13 (12.5%) developed epilepsy with a mean period of 4.2 months during long‐term follow‐up. Among ML models, XGBoost achieved the highest accuracy (0.89) and specificity (0.95) but had low sensitivity (0.46). J48 achieved the highest sensitivity (0.87) and ROC AUC (0.79), with a specificity of 0.71. Incorporating clinical features, including recurrent seizures, increased sensitivity to 0.95. The most relevant predictors were patient history, the frequency band powers particularly increased power in lower frequency bands and HFOs counts. The performance of individual machine learning models in this study also provides interesting comparisons. XGBoost achieved the highest accuracy (0.89) and specificity (0.95); however, its sensitivity remained low (0.46), illustrating the frequent trade‐off between sensitivity and specificity when dealing with imbalanced datasets (He and Garcia, 2009). The J48 Consolidated Decision Tree, despite lower specificity (0.71), showed higher sensitivity (0.87) and a superior ROC AUC score, suggesting it may be more suitable for predicting future epilepsy in this context.

Previous studies assessing the predictive value of EEG after FS have yielded conflicting results, often relying on conventional visual interpretation.^35^ Our approach differs in several key aspects: (i) exclusive use of N2 sleep segments, a stage known for optimal detection of epileptiform discharges and oscillatory biomarkers^36^; (ii) quantitative, automated extraction of time‐domain features, including HFOs, rather than relying solely on visual assessment^37^ and (iii) direct comparison of multiple ML classifiers to identify the most effective predictive model. These methodological differences are crucial when interpreting our findings in the context of existing literature, as earlier works have varied in EEG segment selection, feature extraction techniques, and analytical models.

When compared with previous research, several notable differences emerge.^38^, ^39^ Earlier works often pursued broader objectives—for example, differentiating between multiple seizure types or achieving high overall accuracy in seizure detection—whereas the present study specifically targets the prediction of future epilepsy following a febrile seizure. Additionally, Ayub et al. focused on real‐time detection systems and automated diagnostic pipelines using deep learning architectures, in contrast to our approach, which applied a range of traditional and ensemble machine learning algorithms after preselecting potentially informative EEG features.^39^

In terms of performance, Zhang et al. reported accuracy rates exceeding 90%, whereas our best‐performing model—the J48 Minority Reweighted—achieved an accuracy of 73% and an area under the ROC curve (AUC) of 0.79, with a specificity of 71%.^40^ Importantly, our model achieved a higher sensitivity (87%), indicating a stronger ability to identify children at risk for epilepsy. This higher sensitivity, however, was obtained at the cost of lower specificity, leading to an increased rate of false positives. Such differences may be explained by the rarity of the outcome (only 12.5% of our cohort developed epilepsy) and the methodological choice to analyze EEG data specifically from N2 sleep, a stage known to influence the detectability of epileptiform activity.^40^ These findings underscore that model performance should be interpreted in the context of intended clinical use, as high‐sensitivity models may be preferable for risk stratification, whereas high‐specificity models may be more appropriate for confirmatory assessment.

Finally, this study's findings highlight the significance of including patient history and HFOs as important predictors, a notable contrast to earlier studies that primarily focused on features extracted through frequency analysis and other algorithmic methods. However, the study does not directly compare ML predictions with conventional clinical risk stratification or expert EEG interpretation, and future studies should evaluate the additive value of ML approaches alongside standard clinical assessment. The inclusion of patient history, a clinically relevant variable, underscores the study's attempt to integrate readily available clinical information into the predictive model, improving clinical relevance beyond purely EEG‐based analyses.

The main limitations of the present study are its retrospective design and the relatively small sample size. In addition, the single‐center nature of the cohort may limit demographic and clinical diversity, thereby restricting the external validity of the findings. Furthermore, the absence of external validation using an independent cohort limits the immediate generalizability of the models, and external validation should be considered a prerequisite before clinical implementation.

However, we acknowledge that restricting the analysis to N2 sleep may introduce sampling bias, as some patients may exhibit epileptiform activity, background slowing, or other relevant abnormalities during wakefulness. Consequently, patients whose pathological EEG features were present only in the awake state may have been underrepresented in the present cohort. Although for the patients with FS, age‐related difficulties present in awake EEG recordings. Future studies may incorporate multiple vigilance states, including awake EEG and additional sleep stages, to achieve a more comprehensive electrophysiological characterization and improve generalizability. Nevertheless, other EEG states—particularly post‐nap wakefulness and additional sleep stages—may contain complementary predictive information, which could further enhance model performance in future studies.

Collectively, these limitations highlight the need for cautious interpretation of the findings and support the positioning of this study as a proof‐of‐concept rather than a definitive predictive framework.

In conclusion, this research provides valuable insights into the potential of ML in predicting epilepsy risk following a FS. Initial EEG after the first FS might contribute to prognostication. However, given the retrospective design, limited sample size, and lack of external validation, makes those findings to currently be regarded as hypothesis‐generating rather than practice‐changing. At the same time, the study highlights the complexities inherent in predicting rare outcomes, demonstrating that no single model consistently outperforms others across all performance metrics. Further research with larger datasets, the inclusion of various EEG stages, and potentially longitudinal studies are crucial for refining and validating these models, ultimately leading to better clinical outcomes for children experiencing febrile seizures.

## AUTHOR CONTRIBUTIONS

**Boran Şekeroğlu:** Conceptualization, data curation, formal analysis, methodology, investigation writing – original draft. **Hüseyin Öztoprak:** Conceptualization, investigation, resources, writing – original draft. **Özlem Yayıcı Köken:** Conceptualization, data curation, formal analysis, investigation, methodology, writing – original draft, writing – review & editing. **Hatice Demir:** Data curation, investigation, methodology. **Mehpare Sarı Yanartaş:** Conceptualization, data curation, methodology, writing – review & editing. **Deniz Yılmaz:** Conceptualization, data curation, writing – review & editing. **Burçin Şanlıdağ:** Conceptualization, formal analysis, investigation, methodology, supervision, writing – original draft, writing – review & editing. **Ayşegül Neşe Çıtak Kurt:** Conceptualization, data curation, methodology, resources, supervision, writing – review & editing.

## FUNDING INFORMATION

This research received no specific grant from any funding agency in the public, commercial, or not‐for‐profit sectors.

## CONFLICT OF INTEREST STATEMENT

The authors declare no conflict of interest.

## PATIENT CONSENT

Written informed consent was obtained from the parents or legal guardians of all participants.

**Test yourself**

1. Which EEG‐derived biomarker was identified as one of the most relevant predictors of future epilepsy following a first febrile seizure in this study?
  - Sleep spindle density
  - Alpha‐band asymmetry
  - High‐frequency oscillations (HFOs)
  - Background slowing only
2. Why was N2 sleep specifically selected for EEG feature extraction in this study?
  - It is the longest sleep stage in children
  - It provides stable conditions for detecting epileptiform activity
  - It eliminates motion artifacts completely
  - It is the only stage where HFOs can be detected
3. Which statement best describes the clinical interpretation of the sensitivity–specificity trade‐off observed among the machine learning models?
  - Models with the highest accuracy are always preferred for clinical use
  - High‐specificity models are optimal for early risk stratification
  - High‐sensitivity models may be more suitable for identifying children at risk of future epilepsy
  - Sensitivity and specificity have no clinical relevance in imbalanced datasets

*Answers may be found in the* supporting information.

## Supporting information


Appendix S1.

Appendix S2.

## Data Availability

The data that support the findings of this study are available from the corresponding author upon reasonable request.
