# Supplementary material for: Can epilepsy be predicted after the first febrile seizure? Insights from machine learning of postictal EEG
Source: Epileptic Disord. 2026 Apr 29;28(4):1150–8. doi: 10.1002/epd2.70250 (PMC13499252; doi:10.1002/epd2.70250)
Supplement: Supplementary file 1 [file EPD2-28-1150-s001.docx]

Answers

1. C) High-frequency oscillations (HFOs)

2.B) It provides stable conditions for detecting epileptiform activity

3.C) High-sensitivity models may be more suitable for identifying children at risk of future epilepsy
